# Gfi1 as a regulator of p53 and a therapeutic target for ALL

**DOI:** 10.18632/oncotarget.933

**Published:** 2013-03-18

**Authors:** Cyrus Khandanpour, Tarik Möröy

**Affiliations:** Department of Hematology, Universitätsklinikum Essen, Essen, Germany; Hematopoiesis and Cancer laboratory, Institut de recherches cliniques de Montéal, Université de Montréal, Canada

The transcriptional repressor Gfi1 can be a so-called “oncorequisite” factor that is required for the development and maintenance of lymphoid neoplasia, such as Acute Lymphoblastic Leukemia (ALL), but does not have a direct role in the ontogeny of the disease. The study supporting this role of Gfi1 (Khandanpour C, Phelan J et al., Cancer Cell, 2013, 23(2):200-214) shows that inhibition of Gfi1 cannot only cure mice from ALL but also blocks the expansion of human primary ALL cells. The study concludes that this feature of Gfi1 could be exploited to improve current ALL therapies.

Acute lymphoblastic leukemia/lymphoma is the most common pediatric cancer and the second most common form of acute leukemia in adults. ALL patients are currently treated with chemotherapy and in certain cases with additional irradiation. Moreover, both autologous or allogeneic bone marrow transplantation (BMT) are used to treat ALL, but many patients still relapse, indicating that new treatment regimens are urgently needed. A number of laboratories have accumulated evidence that Gfi1 maybe implicated in the development of T-ALL. For instance, our laboratory has shown that high levels of Gfi1 can accelerate T-cell lymphomagenesis in mice in the presence of other activated oncogenes. In addition, we also found that Gfi1 is required for an undisturbed development of lymphocytes, but Gfi1 knockout mice still have a thymus and at least partially functional B- and T-cells. Finally, data from the public domain and from the laboratory of H. Leighton Grimes, who is a collaborator and coauthor of the study published in Cancer Cell, indicated that Gfi1 is highly expressed in subsets of human T-cell leukemia. With these findings, the question arose, whether Gfi1 may be required for the initiation, maintenance or progression of ALL and if this were the case, whether Gfi1 may represent a potential new therapeutic target.

To test this hypothesis, mouse models were used in the Grimes and Möröy laboratories that develop diseases similar to T-ALL and B-ALL and in which Gfi1 could be deleted either constitutively or conditionally. In all cases, the deletion of Gfi1 not only delayed T-ALL progression, but also abrogated the ability of the leukemic cells to grow in transplanted hosts. However, loss of Gfi1 did not delay tumor initiation in case of B-ALL but also impeded the ability of the B-ALL cells to grow upon transplantation. The most compelling experiment was the conditional ablation of Gfi1 in mice after a T-cell or B-cell lymphoma was established. The deletion of the Gfi1 gene led to tumor regression and clearance of blast cells from blood and to the survival of the animals, whereas the appropriate control mice all died. The most pertinent question was whether this effect could also be seen in human cells. To test this, immune deficient mice (NSG) were transplanted with cells from a T-ALL patient that had been treated but relapsed and succumbed to the disease. The inhibition of Gfi1 was achieved with a morpholino (an antisense reagent) that was applied to the transplanted NSG mice and could indeed significantly inhibit the expansion of the human T-ALL cells. Since morpholinos have previously been used in the clinic to treat other diseases, this experiment showed that inhibition of Gfi1 was not only beneficial but could also be fully applicable in a clinical setting using these antisense reagents.

**Figure 1:**
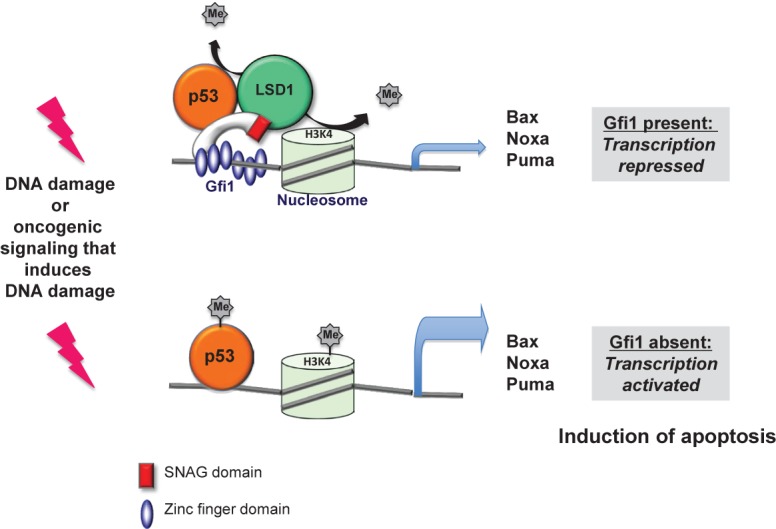
Model of Gfi1 function in T-ALL: p53 dependent transcription of target genes is activated in the absence of Gfi1 DNA damage occurs in tumor cells in the process of malignant transformation according to the concept that oncogenic lesions induce a DNA damage response. As a consequence p53 is activated, but is controlled by Gfi1 because it can recruit LSD1 to p53 to keep it de-methylated at its C-terminus. If Gfi1 is lacking, LSD1 cannot de-methylate p53 with the consequence that p53 is activated and causes the over-expression of apoptosis inducing genes, which eventually leads to the elimination of leukemic cells.

The study also offers an explanation how the ablation of Gf1 may lead to regression of leukemia. It is known that Gfi1 binds to a Lysine specific demethylase (called LSD1) through a N-terminal 20 amino acid domain, which is conserved between Gfi1 and a number of other, similar transcription factors such as Snail and Slug and is thus called SNAG domain. LSD1 can remove methyl groups from histones, typically histone H3, and thereby mediates the transcriptional repressor function of Gfi1. But LSD1 has also other substrates, notably the tumor suppressor p53, which it can demethylate at carboxy-terminal Lysine residues. This demethylation regulates several features of p53 among them its efficiency of chromatin binding. We found that Gfi1 can bind to p53 and that both factors co-occupy common target genes that encode pro-apoptotic proteins such as Puma, Noxa and Bax. Evidence from chromatin-immune-precipitation (Ch-IP) experiments suggests that Gfi1 forms a ternary complex with p53 and LSD1 at p53 target genes such as Puma, Noxa and Bax. Once a DNA damage signal occurs, these target genes become transcriptionally activated and initiate apoptosis. We could show that in leukemic cells, a DNA damage response pathway is active, very likely as a result of ongoing oncogenic signaling in these cells. This was in agreement with a concept that was previously proposed by Jiri Bartek and colleagues who stated that the process of malignant transformation causes DNA damage. We propose that in such a situation, Gfi1 recruits LSD1 to p53 and dampens its activity by de-methylating p53 at C-terminal lysines to prevent immediate apoptosis. In the absence of Gfi1, however, this demethylation cannot occur and p53 is overactive, killing the tumor cells and in our case eliminates the leukemia (Figure 1).

In summary, we have identified Gfi1 as a so-called “oncorequisite” factor, which does not play a direct role in the process of malignant transformation as such, but is critically required by tumor cells to survive and progress, in particular to counteract p53 mediated cell death. Thus, targeting Gfi1 offers a new and very promising approach to treat T-ALL and to improve therapy outcome. We conclude from our data that absence or inhibition of Gfi1 expression sensitizes leukemic cells to undergo accelerated cell death. The curative effect that Gfi1 ablation has on the leukemia outweighs the negative effects that a deletion of Gfi1 will have, such as neutropenia, in particular since these will be transient. This suggests that inhibition of Gfi1 expression could be a new adjuvant therapy that renders current ALL therapies more effective. Studies are currently under way to test this possibility in a clinical setting.

